# Unusual Fusobacterium Nucleatum Infection Presenting as a Liver Abscess Associated With Cephalic Vein Thrombosis

**DOI:** 10.7759/cureus.10971

**Published:** 2020-10-15

**Authors:** Shaylika Chauhan, Vipul Gidwani

**Affiliations:** 1 Internal Medicine, Geisinger Commonwealth School of Medicine, Scranton, USA; 2 Internal Medicine, Parkview Health, Fort Wayne, USA

**Keywords:** fusobacterium nucleatum, liver abscess, emerging pathogen, septic shock

## Abstract

Pyogenic liver abscesses are uncommon entities with potentially devastating consequences requiring immediate diagnosis and treatment. Fusobacterium nucleatum is an anaerobic, gram-negative oral commensal that has been seldom reported as a cause of liver abscess, particularly in immunocompetent hosts. We describe a case of an 80-year-old female patient presenting with a fusobacterium liver abscess associated with thrombosis of the left cephalic vein.

## Introduction

Fusobacterium nucleatum is an emerging pathogen that has recently been implicated in a wide array of medical diseases, including gastrointestinal disorders (colorectal cancer, inflammatory bowel disease, appendicitis), cardiovascular disease, rheumatoid arthritis, respiratory tract infections, Lemierre’s syndrome, organ abscesses, cerebral aneurysm, and Alzheimer’s disease [1].

Fusobacterium nucleatum is an anaerobic gram-negative oral commensal, which while ubiquitous in the oral cavity is absent or infrequently detected elsewhere in the body under normal conditions.

Pyogenic liver abscesses are uncommon entities with potentially devastating consequences requiring immediate diagnosis and treatment. Fusobacterium nucleatum is an anaerobic, gram-negative oral commensal, which has been seldom reported as a cause of liver abscess in immunocompetent hosts. We describe a case of an 80-year-old female patient presenting with a fusobacterium liver abscess associated with thrombosis of the left cephalic vein.

## Case presentation

The patient is an 80-year-old white female who was doing well until five days prior to arrival when she first had mild right back pain. Then over the three days before presenting to the hospital, she began to feel malaise and mild feverishness. On the morning of admission, she felt so bad that she pushed her lifeline button, and the emergency medical services (EMS) arrived to find her lethargic, in pain, and feverish. She was brought to the hospital where she was found to be febrile with a temp of 103 degrees, hypotensive to 88/50 mmHg, with a respiratory rate of 18 in rapid atrial fibrillation, and in acute renal failure. Lab results were as follows:

**Table 1:** Laboratory results WBC: white blood cell; ALT: alanine transaminase; AST: aspartate aminotransferase; ALP: alkaline phosphatase; T.bili: total bilirubin

	Day of admission	Day 4	Day 5 post drain	Day 8 (discharge)
Hemoglobin	13g/dl	10.7g/dl	10.2g/dl	10.3g/dl
WBC	19.7K/ul	34.7K/ul	29.7 K/ul	16.5K/ul
Platelets	180K/ul	148K/ul	155K/ul	281K/ul
Creatinine	2.13mg/dl	2.27mg/dl	2.40mg/dl	2.35mg/dl
ALT	305U/L	240U/L	190U/L	90U/L
AST	270U/L	118U/L	90U/L	33U/L
ALP	188U/L	241U/L	259U/L	179U/L
T.Bili	0.8mg/dl	1mg/dl	1.2mg/dl	0.9mg/dl

**Figure 1:**
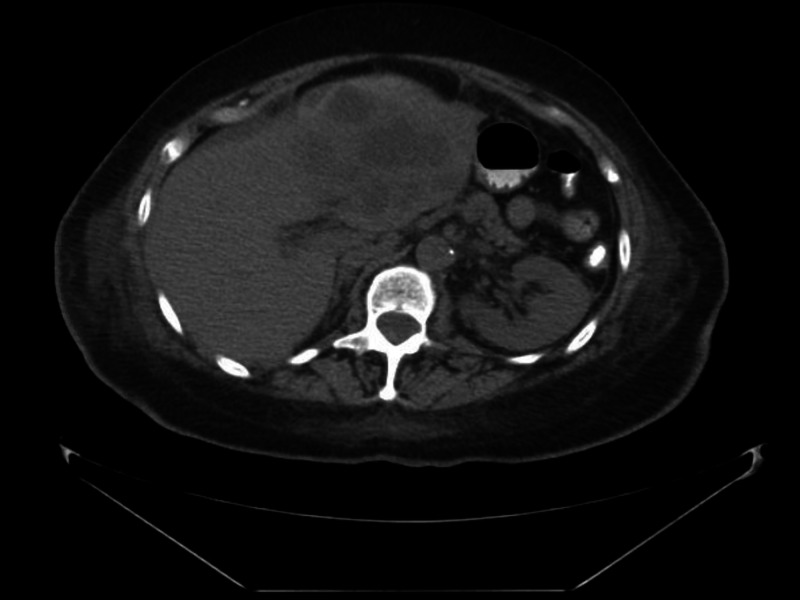
Axial CT images of the abdomen and pelvis w/o contrast demonstrating a solid mass within the left lobe involving segments II, III, and IV measuring 6.7 x 7.8 x 6.5 cm, which could represent primary or metastatic tumor CT: computed tomography

Abdominal CT (Figure 1) revealed a large liver mass, thought to be a tumor. She was admitted to the intensive care unit (ICU), vasopressors were started, and a central line was placed. She was initially treated with piperacillin-tazobactam and vancomycin. Her blood pressure (BP) improved, but her white blood cell (WBC) continued to climb. It was unclear for a few days how the infection related to the liver mass. To determine if it were an abscess, she would need an imaging study with contrast, but her kidneys were at risk and this was not done. Her antibiotics were changed to intravenous (IV) meropenem.

**Figure 2:**
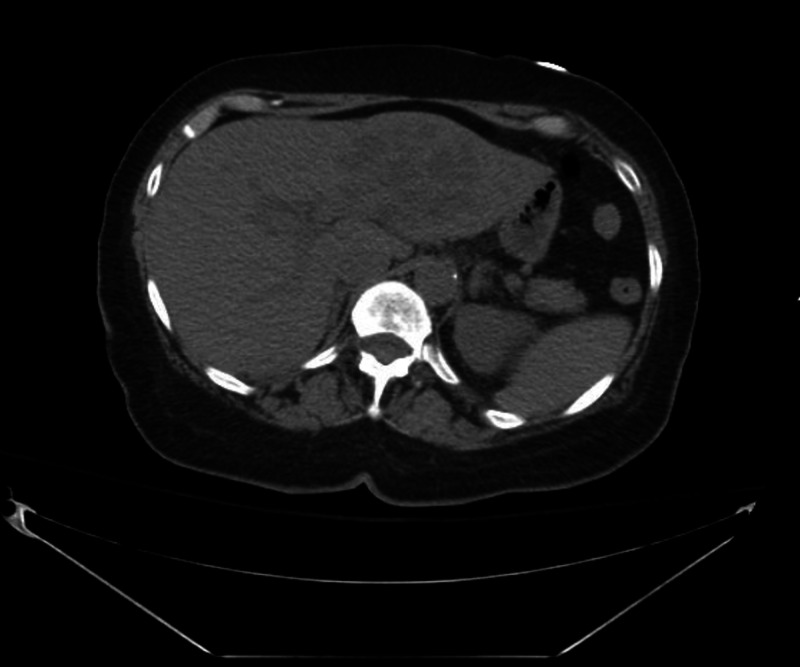
CTA/P showing a significant interval increase in the size of the heterogeneous left liver lesion, which now measures 11.5 cm in maximal dimension as compared with 7.8 cm on the prior CT scan four days earlier The lesion now has a more multiloculated appearance, highly suggestive of an abscess given the rapid interval change. There are new areas of hyperdensity along the inferior aspect of the lesion suggestive of areas of hemorrhage within the abscess. CTA/P: computed tomography arterial portography

On day four of hospitalization, a repeat CT abdomen (Figure 2) was ordered without contrast due to increasing WBCs and an increase in abdominal pain and bloating. It revealed a rapid enlargement of the liver mass, confirming that this was an abscess and not a tumor. A discussion was held with the interventional radiologist and an abdominal drain was placed. Sixty ccs of pus and watery blood were drained from the abscess, but it was found to have many loculations that were difficult to break up.

On day six, the patient complained of shortness of breath at rest. A ventilation-perfusion (VQ) scan and lower-extremity (LE) Doppler were negative for blood clots but D-Dimer was significantly elevated. The blood cultures showed positive for gram-negative rods, later identified as fusobacterium nucleatum, an oral organism associated with thrombi. Venous Doppler of the internal jugular vein and other upper extremity veins was done. Because of a superficial thrombus found in the left cephalic vein at the antecubital level, a peripherally inserted central catheter (PICC) line was placed in the right upper chest wall. Antibiotics were changed to IV rocephin and Flagyl, the latter changed to oral. The patient gradually improved and was then stable for discharge home.

## Discussion

Fusobacterium nucleatum is implicated in periodontal diseases, including gingivitis, chronic periodontitis, localized aggressive periodontitis, and generalized aggressive periodontitis, as also endodontic infections, including pulp necrosis and periapical periodontitis. Smoking [2] and type-2 diabetes mellitus [3] predispose to a higher level of Fusobacterium nucleatum.

Fusobacterium nucleatum is implicated in a broad array of adverse pregnancy outcomes, including chorioamnionitis, preterm premature rupture of membranes, preterm labor, intrauterine growth retardation, low birth weight, stillbirth, and neonatal sepsis [1]. Fusobacterium nucleatum in cord blood is as common as Escherichia coli and Group B streptococcus [4] though not as often checked, pointing to a glaring lacuna in hospital diagnostic assays.

Han et al. reported a case of stillbirth caused by Fusobacterium nucleatum, which likely originated from the mother's pregnancy-associated gingivitis [5]. Subsequent placental pathology showed acute chorioamnionitis with gram stain and placental cultures being positive for Fusobacterium nucleatum. Infants autopsy showed Fusobacterium nucleatum isolated from both the lung and stomach on pure culture. The results of polymerase chain reaction (PCR) and clone analysis indicated that Fusobacterium nucleatum identified in the infant was present in the mother’s subgingival flora but absent from her supragingival, vaginal, or rectal floras. Bacterial translocation from previously unrecognized oral bacteria, translocated to the uterus independent of the ascending vaginal route can explain previously unexplained stillbirths. This underscores the need for expanded microbiological testing as also testing and treatment of periodontal diseases in women to decrease pregnancy morbidity and mortality.

Fusobacterium nucleatum stimulates colorectal cancer growth by modulating the E-cadherin/β catenin signaling via its unique FadA adhesin and potentiates colorectal tumorigenesis in Apcmin/+ mice [5].

A hepatic abscess (HA) can be defined as a suppurated cavity caused by the invasion and multiplication of microorganisms within healthy or diseased liver parenchyma. A hepatic abscess is rare and incidence varies with pyogenic abscess being more common in developed countries and amoebic abscesses in developing countries [6].

Pyogenic liver abscesses are potentially life-threatening, with an estimated incidence rate of 2.3 cases per 100 000 patients. The mortality rate still ranges from 2% to 12% despite rapid clinical advances [7].

A pyogenic liver abscess from Fusobacterium is a rare diagnosis and behaves clinically like any other anaerobic body cavity infections; however, jaundice and right upper quadrant pain are inconsistent findings [8].

Fusobacterium has been shown to have an association with Lemierre’s syndrome, including the development of a pyogenic thrombosis of the internal jugular vein and septic emboli to the lungs, brain, and liver. Lemierre’s syndrome is an uncommon infection, with an annual incidence of between 0.05 and 0.09 per 100,000 population. While our patient didn’t have evidence of Lemierre’s syndrome, she did develop a superficial thrombus in the left cephalic vein [9].

## Conclusions

Fusobacterium nucleatum has been recently found to be implicated in a host of diseases, including colorectal cancer, inflammatory bowel disease, appendicitis, cardiovascular disease, rheumatoid arthritis, respiratory tract infections, Lemierre’s syndrome, organ abscesses, cerebral aneurysm, and Alzheimer’s disease. We have reported an unusual case of a Fusobacterium liver abscess associated with thrombosis of the left cephalic vein. Empiric intravenous antibiotic therapy with metronidazole plus ceftriaxone is the first line and should be continued for four to six weeks. When a liver abscess presents without an evident source, oropharyngeal infections, colonic neoplasms, and infective endocarditis should be ruled out as a seeding source. Prompt drainage of the abscess, along with the rapid initiation of antimicrobial therapy after the accurate identification of Fusobacterium species, with antimicrobial sensitivity testing is the mainstay of treatment since Fusobacterium has varying sensitivity to various antibiotics. The accurate identification of Fusobacterium species is critical not only for taxonomic reasons but also for appropriate treatment of the infection since the susceptibility of different Fusobacterium species to antibiotics varies widely. It is crucial to update microbial detection technologies in clinical practice for accurate diagnosis of the disease and for the identification of individuals at risk.
